# Prospective Validation of a Prediction Model for the Diagnosis of Acute Pancreatitis

**DOI:** 10.1001/jamanetworkopen.2024.19014

**Published:** 2024-06-28

**Authors:** David X. Jin, Ronilda Lacson, Mahsa Eskian, Julia McNabb-Baltar, Peter A. Banks, Stephanie R. Kayden, Ali S. Raja, Ramin Khorasani

**Affiliations:** 1 Center for Pancreatic Disease, Division of Gastroenterology, Hepatology, and Endoscopy, Department of Medicine, Brigham and Women’s Hospital, Harvard Medical School, Boston, Massachusetts; 2 Center for Evidence-Based Imaging, Department of Radiology, Brigham and Women’s Hospital, Harvard Medical School, Boston, Massachusetts; 3 Department of Emergency Medicine, Brigham and Women’s Hospital, Harvard Medical School, Boston, Massachusetts; 4 Department of Emergency Medicine, Massachusetts General Hospital, Harvard Medical School, Boston

## Abstract

**Importance:**

While most patients with acute pancreatitis (AP) fulfill diagnostic criteria with characteristic abdominal pain and serum lipase levels of at least 3 times the upper limit of normal (reference range) at presentation, early imaging is often used for confirmation. A prior prediction model and corresponding point-based score were developed using nonimaging parameters to diagnose AP in patients presenting to the emergency department (ED).

**Objective:**

To evaluate the performance of the prediction model to diagnose AP in a prospective patient cohort.

**Design, Setting, and Participants:**

This prospective diagnostic study included consecutive adult patients presenting to the ED between January 1, 2020, and March 9, 2021, at 2 large academic medical centers in the northeastern US with serum lipase levels at least 3 times the upper limit of normal. Patients transferred from outside institutions or with malignant disease and established intra-abdominal metastases, acute trauma, or altered mentation were excluded. Data were analyzed from October 15 to October 23, 2023.

**Exposures:**

Participants were assigned scores for initial serum lipase level, number of prior AP episodes, prior cholelithiasis, abdominal surgery within 2 months, presence of epigastric pain, pain of worsening severity, duration from pain onset to presentation, and pain level at ED presentation.

**Main Outcome and Measures:**

A final diagnosis of AP, established by expert review of hospitalization records.

**Results:**

Prospective scores in 349 participants (mean [SD] age, 53.0 [18.8] years; 184 women [52.7%]; 66 Black [18.9%]; 199 White [57.0%]) demonstrated an area under the receiver operating characteristics curve of 0.91. A score of at least 6 points achieved highest accuracy (*F* score, 82.0), corresponding to a sensitivity of 81.5%, specificity of 85.9%, positive predictive value of 82.6%, and negative predictive value of 85.1% for AP diagnosis. Early computed tomography or magnetic resonance imaging was performed more often in participants predicted to have AP (116 of 155 [74.8%] with a score ≥6 vs 111 of 194 [57.2%] with a score <6; *P* < .001). Early imaging revealed an alternative diagnosis in 8 of 116 participants (6.9%) with scores of at least 6 points, 1 of 93 (1.1%) with scores of at least 7 points, and 1 of 73 (1.4%) with scores of at least 8 points.

**Conclusions and Relevance:**

In this multicenter diagnostic study, the prediction model demonstrated excellent AP diagnostic accuracy. Its application may be used to avoid unnecessary confirmatory imaging.

## Key Points

**Question:**

Does a novel prediction model that uses 8 nonimaging parameters diagnose acute pancreatitis in patients presenting to the emergency department with elevated serum lipase levels?

**Findings:**

In this diagnostic study validating a prediction model, the model demonstrated excellent accuracy. At a score of at least 6 points, prediction model accuracy and performance were optimized, serious alternative diagnoses were uncommon, and diagnostic yield of early imaging was low.

**Meaning:**

This prediction model may accurately diagnose acute pancreatitis and obviate the need for confirmatory diagnostic imaging in many patients.

## Teaser

This diagnostic study evaluates the performance of a prediction model of acute pancreatitis in a prospective cohort of patients presenting to the emergency department with serum lipase levels elevated to 3 times the upper limit of the reference range.

## Introduction

The diagnosis of acute pancreatitis (AP) currently requires fulfilling at least 2 of 3 criteria: (1) characteristic abdominal pain, (2) serum lipase (or amylase) levels elevated to at least 3 times the upper limit of normal (or reference range) (ULN), and (3) characteristic findings on imaging, typically abdominal computed tomography (CT) or magnetic resonance imaging (MRI).^1^ While 90% of patients fulfill symptom and laboratory criteria at presentation and therefore do not require imaging confirmation, early imaging remains overused.^2^

To address this limitation, a prediction model and corresponding point-based score were previously developed to diagnose AP in patients presenting to the emergency department (ED) with suspected AP, defined as serum lipase levels of at least 3 times the ULN.^3^ This model used 8 nonimaging parameters found to be most associated with AP, each readily ascertainable at presentation: 1 laboratory variable (initial serum lipase level), 3 variables pertaining to medical history (number of prior AP episodes, prior cholelithiasis, or recent abdominal surgery), and 4 variables characterizing abdominal pain (pain localized to the epigastrium, pain of worsening severity, duration from pain onset to presentation, and pain level at ED presentation).

While the model demonstrated excellent discriminatory accuracy for AP in a retrospective cohort, it has not been prospectively validated.^3^ The objective of this study was to evaluate the performance of the prediction model to diagnose AP when applied to a prospective cohort of patients.

## Methods

### Study Design, Setting, and Participants

This prospective diagnostic study followed the 2015 Transparent Reporting of a Multivariable Prediction Model for Individual Prognosis or Diagnosis (TRIPOD) guidelines. This study was conducted at 2 large academic medical centers in the northeastern US, Brigham and Women’s Hospital (BWH), Boston, Massachusetts, and Massachusetts General Hospital (MGH), Boston, and was approved by the institutional review board of each hospital with waiver of the requirement for informed consent owing to the use of deidentified data. The data-gathering period was from January 1, 2020, to March 9, 2021. Daily consecutive cases of hyperlipasemia, defined as serum lipase levels of at least 3 times the ULN (≥180 U/L), were identified using an ED laboratory query and were eligible for inclusion. Patients transferred from outside hospitals, with malignant disease and known intra-abdominal metastases, with acute traumatic injury, or with altered mentation precluding accurate history taking were excluded when deriving the original model^3^ and similarly excluded from this validation study.

### Primary Outcome

The primary outcome was a diagnosis of AP according to the current reference standard,^1^ determined by an experienced medical pancreatologist (D.X.J.) after a comprehensive electronic health record (EHR) review of the ED course and, when applicable, hospitalization and postdischarge course. This investigator was blinded to the final score of each participant. Because all included participants presented with serum lipase levels of at least 3 times the ULN, AP was diagnosed if (1) CT or MRI revealed findings characteristic of AP at any time during the hospitalization or 3-month postdischarge period, or (2) presenting symptoms were characteristic of AP and not clearly explained by another condition. Alternative diagnoses were recorded, when applicable, by the same investigator.

### Prediction Model and Data Collection

Table 1 presents the point-based scoring model.^3^ In this study, data for each variable in the model were prospectively extracted from the EHR by a separate study investigator (M.E.) blinded to the final diagnosis. Data regarding baseline demographic characteristics, including sex and race and ethnicity were participant defined and were included to reflect the diversity of the study population. Variables pertaining to medical history and presenting symptoms not documented in the EHR were treated implicitly as absent. The initial recorded pain score (0-10) in the ED was used for pain severity. Discrepant variables among different clinicians were managed by prioritizing documentation from the gastrointestinal consultant (when applicable), the admitting medical or surgical clinician, and the ED clinician. Points assigned to each variable were summed to create a final score ranging from −3 to 14.

**Table 1.** Prediction Model for the Diagnosis of AP: Categories and Points

Parameter	Points
No. of prior AP episodes	
0	0
1	1
2	2
3	3
≥4	4
History of cholelithiasis	
No	0
Yes	2
Abdominal surgery in prior 2 mo	
No	0
Yes	−2
Symptoms	
Epigastric pain	
No	0
Yes	2
Worsening severity	
No	0
Yes	1
Duration of pain, d	
<5	0
≥5	−1
Pain severity (of 10)	
Mild (0-3)	0
Moderate (4-6)	2
Severe (7-10)	3
Serum lipase level	
≥3 to <10 times the ULN	0
≥10 to <20 times the ULN	1
≥20 times the ULN	2

Abbreviations: AP, acute pancreatitis; ULN, upper limit of normal (reference range).

Use of early imaging, defined as abdominal CT or MRI performed within 24 hours of ED presentation, was recorded. In participants without AP, the diagnostic yield of early imaging was noted.

### Sample Size Considerations

Prior application of the model to a retrospective cohort with an AP prevalence of 50%^3^ found that a diagnostic score of at least 6 points was 77% sensitive and 92% specific for AP. As such, a sample size of 137 participants was calculated (precision, ±0.1; α = .05) for each site for this validation study.

### Model Validation

Discriminatory accuracy was estimated by calculating areas under the receiver operating characteristics curve (AUROC). Diagnostic performance was assessed by calculating the sensitivity, specificity, positive predictive value (PPV), and negative predictive value (NPV) at different diagnostic point cutoffs. The cutoff that maximized accuracy and diagnostic performance was determined by calculating *F* scores and Youden indices.^4^ The *F* score calculates the harmonic mean between sensitivity and PPV. In contrast, the Youden index calculates the optimal diagnostic threshold, which maximizes the difference between the true- and false-positive rates.

### Statistical Analysis

Data were analyzed from October 15 to 23, 2023. Results are expressed as the number (percentage) for categorical variables and as the mean (SD) or median (IQR) for continuous variables. A χ^2^ test was used for comparisons between categorical variables. A 2-tailed *P* < .05 was considered statistically significant. Data analysis was performed using R, version 2.8.0 (R Project for Statistical Computing).

## Results

### Study Population

**Figure 1.**
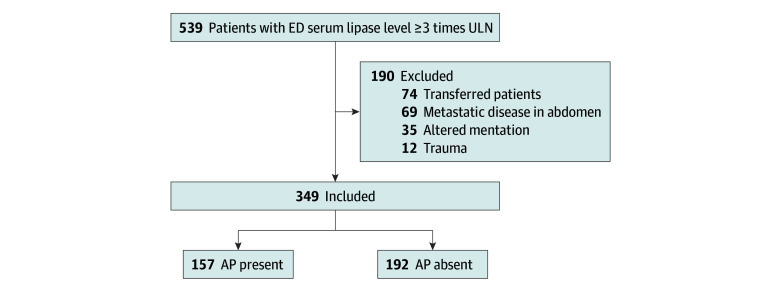
Flow Diagram of Study Participants AP indicates acute pancreatitis; ED, emergency department; and ULN, upper limit of normal (reference range).

A total of 349 participants fulfilled the inclusion criteria (207 at BWH and 142 at MGH) and were included in the prospective validation cohort (Figure 1). Of these, 165 patients (47.3%) were men and 184 (52.7%) were women; mean (SD) age was 53.0 (18.8) years. In terms of race and ethnicity, 19 patients (5.4%) were Asian, 66 (18.9%) were Black, 17 (4.9%) were Hispanic, 199 (57.0%) were White, 38 (10.9%) were of other race or ethnicity, and 10 (2.9%) were of unknown race or ethnicity. Table 2 presents participant demographic and model-specific characteristics.

**Table 2.** Characteristics of Patients Seen in the Emergency Department With Suspected AP

Characteristic	Values (N = 349)^a^
Age, y	
Mean (SD)	53.0 (18.8)
Median (IQR)	52 (37-68)
Sex	
Male	165 (47.3)
Female	184 (52.7)
Race and ethnicity	
Asian	19 (5.4)
Black	66 (18.9)
Hispanic	17 (4.9)
White	199 (57.0)
Other^b^	38 (10.9)
Declined or unavailable	10 (2.9)
Total AP	157 (45.0)
Model parameters	
Serum lipase level	
≥3 to <10 times the ULN	181 (51.9)
≥10 to <20 times the ULN	39 (11.2)
≥20 times the ULN	129 (37.0)
Epigastric pain	170 (48.7)
Worsening severity	54 (15.5)
Time to presentation, d	
Mean (SD)	3.6 (7.7)
Median (IQR)	1 (0-3)
Pain severity (of 10)	
Mild (0-3)	115 (33.0)
Moderate (4-6)	53 (15.2)
Severe (7-10)	181 (51.9)
No. of prior AP episodes	
Mean (SD)	0.5 (1.0)
Median (IQR)	0 (0-1)
History of cholelithiasis	80 (22.9)
Abdominal surgery in prior 2 mo	27 (7.7)

Abbreviations: AP, acute pancreatitis; ULN, upper limit of normal (reference range).

^a^ Unless otherwise indicated, data are expressed as No. (%) of patients. Percentages have been rounded and may not total 100.

^b^ Includes all other responses not included in the categories above.

### Incidence of AP and Alternative Diagnoses

Of the final cohort, 157 participants (45.0%) were determined to have a final diagnosis of AP. The most common non-AP diagnoses (n = 192) were nausea, vomiting, and gastroenteritis (35 [18.2%]), kidney failure (24 [12.5%]), alcohol intoxication (17 [8.9%]), recent surgery (14 [7.3%]), acute hepatitis or cirrhosis (14 [7.3%]), small-bowel obstruction (13 [6.8%]), and biliary tract disease (12 [6.3%]).

### Model Performance

The median score of the entire cohort was 5 (range, −3 to 14) points. Table 3 presents the diagnostic performance of the model at cutoff scores of at least 5, at least 6, at least 7, and at least 8 points. The *F* score (82.0) and Youden index (0.674) were maximized at a diagnostic cutoff of at least 6 points. At this cutoff, which was met in 155 participants (44.4%), the model performed with sensitivity of 81.5%, specificity of 85.9%, PPV of 82.6%, and NPV of 85.1%. At a more conservative diagnostic cutoff of at least 8 points, which was met in 96 participants (27.5%), specificity (96.4%) and PPV (92.7%) were gained at the expense of lower sensitivity (56.7%) and NPV (73.1%).

**Table 3.** Diagnostic Performance at Various Point Score Thresholds

Risk threshold	Diagnostic performance^a^
**≥5 Points**	
Sensitivity	134/157 (85.4)
Specificity	148/192 (77.1)
PPV	134/178 (75.3)
NPV	148/171 (86.5)
*F* score	80.0
Youden index	0.625
**≥6 Points**	
Sensitivity	128/157 (81.5)
Specificity	165/192 (85.9)
PPV	128/155 (82.6)
NPV	165/194 (85.1)
*F* score	82.0
Youden index	0.674
**≥7 Points**	
Sensitivity	112/157 (71.3)
Specificity	181/192 (94.3)
PPV	112/123 (91.1)
NPV	181/226 (80.1)
*F* score	80.0
Youden index	0.656
**≥8 Points**	
Sensitivity	89/157 (56.7)
Specificity	185/192 (96.4)
PPV	89/96 (92.7)
NPV	185/253 (73.1)
*F* score	70.4
Youden index	0.531

Abbreviations: NPV, negative predictive value; PPV, positive predictive value.

^a^ Unless otherwise indicated, data are expressed as No./total No. (%) of patients.

**Figure 2.**
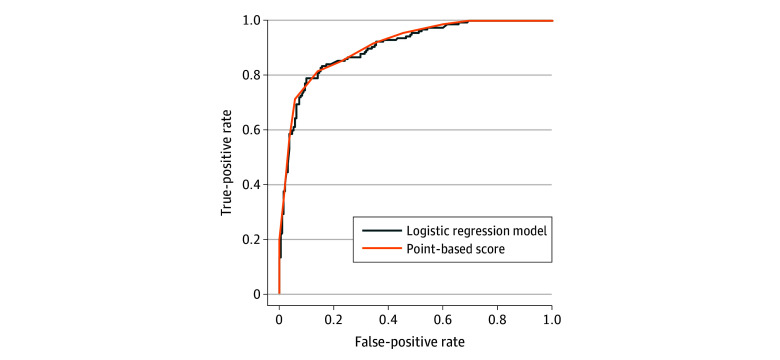
Area Under the Receiver Operating Characteristics Curve for the Diagnosis of Acute Pancreatitis The area under the receiver operating characteristics curve was 0.90 for the logistic regression model and 0.91 for the point-based score.

Figure 2 presents the receiver operating characteristics curves for the logistic regression and point-based score when applied to the prospective validation cohort. The AUROC was 0.90 for the regression model and 0.91 for the point-based score.

### Use, Timing, and Yield of Early Diagnostic Imaging

Abdominal CT or MRI was performed early (within 24 hours of presentation) in 227 participants (65.0%), and at any point during the hospitalization in 251 (71.9%); 98 (28.1%) received no imaging. Early imaging was performed more often in those predicted to have AP (116 of 155 [74.8%] when the score was ≥6 vs 111 of 194 [57.2%] when the score was <6; *P* < .001). Among those undergoing early imaging, the median time from return of serum lipase results to imaging performed was 86 (IQR, 35-175) minutes. Early imaging was performed before lipase results were returned in 27 participants (11.9%). Among participants with a score of at least 6 points, early imaging was performed in 116 (representing 51.1% of early imaging). Of these 116 early imaging studies, 8 (6.9%) revealed an alternative diagnosis (3 cases of enteritis or colitis, 3 cases of cholangitis or choledocholithiasis, 1 case of diverticulitis, and 1 case of a duodenal hematoma). Among patients with a score of at least 7 points, early imaging was performed in 93 (representing 41.0% of early imaging). Of these 93 early imaging studies, 1 MRI (1.1%) revealed choledocholithiasis without AP. Among participants with a score of at least 8 points, early imaging was performed in 73 (representing 32.2% of early imaging). Of these 73 early imaging studies, 1 MRI (1.4%) revealed an alternative diagnosis (the aforementioned choledocholithiasis without AP). The remaining 72 studies (98.6%) revealed AP or were nondiagnostic.

### Serious Alternative Diagnoses

Particular attention was placed on alternative diagnoses, which may elevate serum lipase levels, mimic AP, and require urgent management. There were 13 cases of bowel obstruction (3.7% of the entire cohort). The median score was 3 (range, −3 to 8 points). Only 1 case (0.3% of the entire cohort) scored at least 6 points (8 points): a woman in her 50s who had a history of recurrent small-bowel obstruction due to adhesions, often in the setting of elevated serum lipase levels. During this particular episode, she presented with similar pain, an initial serum lipase level of 1378 U/L (ULN, 60 U/L), and did not undergo imaging due to the recurrent nature of her symptoms. Her pain resolved quickly, and she was discharged following a 2-day hospitalization.

There were 3 cases of bowel perforation (0.9% of the entire cohort). The scores ranged from 2 to 5 points. All 3 cases underwent early CT imaging to confirm the diagnosis. There were no cases of mesenteric ischemia.

## Discussion

In this multicenter study, we prospectively validated a prediction model for the diagnosis of AP among patients presenting to the ED with elevated serum lipase levels. Using nonimaging parameters readily ascertainable at presentation, the model demonstrated excellent discriminatory accuracy (AUROC, 0.91). At a cutoff of at least 6 points, which optimized diagnostic accuracy and performance, the yield of early abdominal CT or MRI was low. This novel model has important implications in the diagnosis of AP.

Acute pancreatitis ranks among the leading contributors to hospitalizations, readmissions, and health care costs among all gastrointestinal tract disorders in the US.^5^ Despite improvements in mortality and length of hospital stay, the cost of hospitalization continues to increase, partly due to increasing reliance on imaging studies, particularly at presentation.^6,7^

The current AP diagnostic criteria, which were first proposed in 2006^8^ and agreed on by international consensus in 2012,^1^ require 2 of the following 3 features: (1) characteristic abdominal pain, (2) serum lipase (or amylase) level of at least 3 times the ULN, and (3) characteristic findings of AP on contrast-enhanced CT or MRI. While multiple AP clinical guidelines^9,10,11^ explicitly state that routine early imaging is not necessary because it rarely reveals alternative diagnoses,^12^ changes clinical management,^13^ or improves outcomes,^6^ we found in this study that early imaging was used in 65.0% of participants.

The reliance on early imaging in AP may be attributed to limitations of the current diagnostic criteria.^1^ For example, how one defines characteristic abdominal pain remains subjective; higher serum lipase level cutoffs are known to have increased PPV^14,15^; and CT and MRI are subject to differences in sensitivity and interobserver agreement.^16,17^ Notably, these 3 diagnostic criteria are weighted equally, yet few studies have compared their diagnostic performance to each other or as a group.^18^

This novel prediction model improves on several of these limitations. First, it provides an objective framework for what constitutes characteristic abdominal pain, described initially as acute onset of persistent, severe, epigastric pain often radiating to the back.^1^ The model assigns points for not only epigastric pain but for shorter duration from pain onset to ED presentation (<5 days) and higher pain scores. Pain radiating to the back was not found to be adequately predictive.^3^ Second, it assigns more points for elevations in serum lipase levels beyond at least 3 times the ULN, which are more specific for differentiating AP from nonpancreatic hyperlipasemia.^14,19,20,21^ Third, it incorporates perhaps the 2 most significant risk factors for AP: a history of AP or choledocholithiasis.^19^

Notably, the model has the potential to obviate the need for confirmatory imaging in many patients who present with hyperlipasemia. Serum lipase measurement is often used in the ED as part of a broad evaluation for nonspecific abdominal pain.^19^ Though commonly associated with AP, hyperlipasemia can develop in a variety of pancreatic and nonpancreatic disorders.^19,20,21^ While many of these, such as cholangitis, decompensated cirrhosis, and kidney failure, can be readily distinguished with history taking and laboratory testing, a few, in particular, may lead to rapid clinical deterioration if not diagnosed promptly: bowel obstruction, bowel ischemia, and visceral perforation. We have prospectively demonstrated that the prediction model is helpful in ruling out these serious alternative diagnoses, which had a prevalence of 4.6% (13 bowel obstruction and 3 bowel perforation diagnoses) in our study population. Using a diagnostic cutoff of at least 6 points, only 1 participant (0.3% of the entire cohort) with a serious alternative diagnosis (small bowel obstruction) would have been misdiagnosed as having AP. The yield of early imaging was similarly low. In the same cohort of participants predicted to have AP (≥6 points), alternative diagnoses were discovered in only 6.9% of early imaging examinations, none of which were immediately life-threatening. At a more conservative diagnostic cutoff of at least 8 points (PPV, 92.7%), only 1.4% of early imaging studies (1 participant with choledocholithiasis) revealed an alternative diagnosis. This highlights the model’s adaptability when operationalized: applying a diagnostic cutoff of at least 6 points for patients with mild symptoms and without organ dysfunction would conceptually have reduced early imaging by 51%. Applying a cutoff of at least 8 points for patients with more severe symptoms and/or signs could further curtail serious misdiagnoses by exploiting a high PPV but still conceptually have reduced early imaging by 32%. Immediate early imaging would continue to be appropriate when there is concern regarding a life-threatening alternative diagnosis.

In addition to diagnostic confirmation, imaging remains a valuable tool to assess for local AP complications such as (peri)pancreatic fluid collections or necrosis, pseudoaneurysmal bleeding, or gastric outlet obstruction. These complications typically develop later in the disease course with persistent or recurrent abdominal pain, increasing organ dysfunction, or intolerance to oral feeding.^1^ Imaging is also helpful when elucidating AP etiology, particularly during the index hospitalization for biliary AP, or after discharge in those with initially idiopathic AP. Future studies could evaluate whether application of the model would alter use of both early and total imaging.

### Strengths and Limitations

The strengths of this study design are that participants were prospectively identified at 2 centers, judiciously evaluated for clinical parameters, and comprehensively reviewed for the presence or absence of AP. This allowed for complete identification of the target cohort and internal and external validation of the model. In addition, we ensured that the sample size was adequate for measurements of diagnostic accuracy.

This study has several limitations. All data were extracted from the EHR rather than directly from the patient. As such, when symptoms were not documented, such as pain of worsening severity, they were implicitly treated as absent. Future prospective studies should focus on model validation at the point of care to limit missing data. The cohort used to derive the original model and for validation in the current study were patients presenting with serum lipase levels of at least 3 times the ULN. However, up to 20% of patients with AP do not meet this lipase threshold and would therefore require imaging for diagnostic confirmation.^15^ Nevertheless, serum lipase measurement is widely used as the initial screening tool for AP, and patients presenting with hyperlipasemia represent a practical cohort for studying model performance. Abdominal CT or MRI imaging was not performed at any point during the hospitalization in 98 patients (28.1%). It is possible that alternative diagnoses could have been found in these patients and therefore the true yield of imaging remains unknown. Both BWH and MGH are large urban academic medical referral centers in the northeastern US. While transferred patients were excluded, future studies should focus on generalizability to community hospitals and geographically diverse patient populations.

## Conclusions

In this multicenter diagnostic study of ED patients presenting with elevated serum lipase levels, the prediction model demonstrated excellent discriminatory accuracy for AP. Future prospective studies are needed to assess whether application of this tool would substantially reduce early diagnostic imaging.

## Notes

Supplement.
Data Sharing Statement
