# Supplementary material for: Prospective Validation of a Prediction Model for the Diagnosis of Acute Pancreatitis
Source: JAMA Netw Open. 2024 Jun 28;7(6):e2419014. doi: 10.1001/jamanetworkopen.2024.19014 (PMC11214112; doi:10.1001/jamanetworkopen.2024.19014)
Supplement: Supplement. — Data Sharing Statement [file jamanetwopen-e2419014-s001.pdf]

Data Sharing Statement

Jin. Prospective Validation of a Prediction Model for the Diagnosis of Acute Pancreatitis. *JAMA Netw Open*. Published June 28, 2024. doi:10.1001/jamanetworkopen.2024.19014

Data
Data available: No
